# Targeting cariogenic virulence of *Streptococcus mutans* by pinosylvin: a promising anti-cariogenic natural agent

**DOI:** 10.3389/fmicb.2026.1911157

**Published:** 2026-10-14

**Authors:** Jingyan Shu, Wei Liu, Yi Qiu, Xinyu Liao, Yuxuan Xiong, Xiang Xie, Yi Xu, Yangyutong Liu, Ying Liu, Jiakuan Peng, Xue Mei

**Affiliations:** 1 Department of Stomatology, Affiliated Hospital of North Sichuan Medical College, Nanchong, Sichuan, China; 2 School of Stomatology, North Sichuan Medical College, Nanchong, Sichuan, China; 3 School of Clinical Medicine, North Sichuan Medical College, Nanchong, Sichuan, China; 4 School of Basic Medical Sciences and Law, North Sichuan Medical College, Nanchong, Sichuan, China; 5 School of Medical Imaging, North Sichuan Medical College, Nanchong, Sichuan, China; 6 College of Pharmacy, North Sichuan Medical College, Nanchong, Sichuan, China; 7 Biotechnology Innovation Drug Application and Transformation Key Laboratory of Sichuan Province, North Sichuan Medical College, Nanchong, Sichuan, China

**Keywords:** biofilm, dental caries, glucosyltransferases, natural products, pinosylvin, *Streptococcus mutans*

## Abstract

Dental caries continues to be a serious worldwide public health issue, with *Streptococcus mutans* (*S. mutans*) identified as the primary etiological pathogen. This study aimed to evaluate the anti-cariogenic potential of pinosylvin (PIN) against *S. mutans*. A combination of *in vitro* and *in vitro* experiments was performed to characterize the anti-cariogenic effects of PIN. *In vitro* assays were conducted to determine the impacts of PIN on the growth, bacterial adhesion, biofilm formation, and lactic acid synthesis of *S. mutans*. Quantitative real-time PCR (qRT-PCR) was applied to quantify the transcript abundance of key cariogenic virulence genes, including *gtfB, gtfC*, and *gtfD*. A rat dental caries model was adopted to assess the biosafety and *in vitro* anti-cariogenic efficacy of PIN. PIN inhibited the growth, bacterial adhesion, biofilm formation, and lactic acid synthesis of *S. mutans*. Consistent with these phenotypic alterations, qRT-PCR analysis demonstrated that PIN downregulated the expression of core cariogenic virulence genes *gtfB, gtfC*, and *gtfD*. *In vivo*, PIN exhibited favorable biocompatibility and anti-cariogenic effects in the rat caries model. Collectively, these findings suggest that PIN exerts anti-cariogenic activity against *S. mutans* by suppressing multiple virulence-associated phenotypes and downregulating key cariogenic genes. The *in vivo* findings further support its biocompatibility and potential anti-cariogenic effect. Nevertheless, the precise molecular mechanisms underlying these effects and suitable delivery strategies of PIN remain to be clarified in future studies.

## 1 Introduction

Dental caries is a prevalent chronic oral disease that poses a severe threat to global public health (Benzian and Beltrán-Aguilar, 2025). The sequelae of dental caries extend beyond oral health, profoundly affecting quality of life by inducing orofacial pain, compromising nutritional intake due to masticatory limitations, and fostering social apprehension, thus diminishing psychosocial well-being (Kurt et al., 2025; Barnes et al., 2026; Ma et al., 2026). The disease primarily results from an imbalance in the local microecology of dental plaque, with *Streptococcus mutans* (*S. mutans*) being the primary cariogenic pathogen (Lemos et al., 2019; Mazurel et al., 2025). In addition to consuming sucrose, glucose, and fructose for glycolysis to generate energy and produce massive lactic acid that induces enamel demineralization, *S. mutans* adheres to and colonizes the enamel surface to initiate caries development (Lin et al., 2021). In this process, *S. mutans* secretes glucosyltransferases (Gtfs) to catalyze the synthesis of different types of glucans from sucrose. These glucans act as a scaffold for *S. mutans* and other oral microorganisms to adhere to and form the extracellular polymeric substance (EPS) (Gao et al., 2023; Atta et al., 2024), further facilitating plaque formation. Compared with planktonic bacteria, those growing in biofilms exhibit enhanced resistance to the host’s immune response and have stronger resistance to antibiotics, biocides, and hydrodynamic shear forces. Their drug resistance can increase by 10 to 1,000-fold, rendering antibiotics ineffective and exacerbating bacterial drug resistance (Davies, 2003; Sharma et al., 2019). Therefore, cariogenic virulence factors of *S. mutans*, such as adhesion, acid production, and EPS, are critical for dental caries.

Currently, routine clinical strategies for caries control mainly include mechanical plaque removal, topical fluoride application and broad-spectrum antibiotic administration. Nevertheless, these conventional interventions encounter prominent clinical limitations (Gloria-Garza et al., 2025). Mechanical cleaning fails to inhibit the rapid recolonization of pathogenic bacteria on tooth surfaces (Hernández et al., 2022). Repeated fluoride exposure induces bacterial adaptive tolerance and further fluoride resistance (Liao et al., 2017). Long-term antibiotic use disrupts oral flora homeostasis and increases the risk of local problems (such as pulpal and periapical illnesses) (Peng et al., 2022). Critically, the overuse of conventional antimicrobials imposes intense selective pressure on oral bacteria (Darby et al., 2023). Therefore, it is critical to develop alternative therapies that precisely inhibit *S. mutans*’ cariogenic factors without causing drug resistance.

Natural compounds are increasingly regarded as attractive research subjects for developing new antimicrobial strategies, owing to their widespread presence in nature and biosafety (Cao et al., 2024; Zheng et al., 2026). In addition, natural compounds can also reduce resistance to traditional antimicrobial agents, providing a way to overcome antibiotic resistance (Zhou J.-F. et al., 2025). Pinosylvin (PIN), a typical stilbenoid polyphenol, isolated from the heartwood of *Pinus* species (e.g., European red pine) (Bakrim et al., 2022), is a natural active small molecule with favorable research and application prospects. Compared with the extensively studied natural anti-caries compounds, including resveratrol, epigallocatechin gallate (EGCG), and curcumin, PIN exhibits certain advantages in molecular structure and physicochemical properties. These conventional anti-caries compounds possess certain limitations, such as poor photostability, high susceptibility to aqueous oxidative degradation, and evident tooth discoloration, which largely restrict their clinical translation (Bianchi et al., 2011; Dinu et al., 2024; Kashi et al., 2025). As a natural bioactive molecule, PIN lacks the 4′-hydroxyl group in its molecular structure, which theoretically confers superior photo-oxidative stability and resistance to oxidative degradation (Drábiková et al., 2012). Moreover, its inherent physicochemical characteristics do not induce tooth discoloration. Previous studies have shown that PIN exerts multiple pharmacological activities, including anti-inflammatory, antioxidant, and anti-tumor effects (Periferakis et al., 2025), and it can inhibit a variety of pathogenic bacteria by disrupting bacterial membranes and interfering with microbial metabolism (Lee et al., 2005). Nevertheless, whether PIN can affect the cariogenic virulence of oral *S. mutans* remains unclear and needs further experimental exploration.

Given the certain limitations of current conventional anti-caries strategies, this study investigated the inhibitory effects of PIN on biofilm formation and key virulence phenotypes of *S. mutans in vitro*, and established a rat caries model to verify its biosafety and caries-preventive efficacy *in vivo,* to provide experimental evidence for further study of this natural compound’s anti-cariogenic properties.

## 2 Materials and methods

### 2.1 Bacterial strains and reagents

The *S. mutans* UA159 strain used in this study was preserved in our laboratory. *S. mutans* UA159 was cultured overnight from a single colony inoculum in brain-heart infusion (BHI) broth (MedChemExpress, Shanghai, China) at 37 °C under anaerobic conditions. BHI supplemented with 1% (w/v) sucrose (BHIS) was used for biofilm formation assays. 0.12% Chlorhexidine (CHX) was applied as the positive control for *in vitro* experiments in this study, which was purchased from MedChemExpress, Shanghai, China. Pinosylvin (PIN, ≥98% HPLC purity; Herpurify Biotechnology, Chengdu, China) was dissolved in dimethyl sulfoxide (DMSO; Beyotime, Shanghai, China) to prepare a stock solution.

### 2.2 Bacterial susceptibility assay

According to the Clinical and Laboratory Standards Institute guidelines (Clinical and Laboratory Standards Institute, 2024), the minimum inhibitory concentration (MIC) of PIN for *S. mutans* was determined using broth microdilution (Zhou et al., 2024). Briefly, *S. mutans* were adjusted to 1 × 10^6^ CFU/mL. PIN was serially two-fold diluted in BHI broth. Equal volumes (100 μL) of bacterial suspension and gradient PIN diluents were mixed in 96-well round-bottom microplates, yielding final PIN concentrations ranging from 2 to 2048 μg/mL. Negative controls were DMSO-containing wells (final concentration 0.5%, v/v). After plates were incubated for 24 h at 37 °C in an anaerobic atmosphere, MIC was identified as the lowest concentration with no visible turbidity. For MBC determination, bacterial suspensions from non-turbid PIN-treated wells were spread on fresh BHI agar plates and incubated anaerobically for 48 h. MBC was defined as the lowest PIN concentration with no bacterial colony formation.

### 2.3 Growth kinetic analysis

*S. mutans* were diluted in fresh BHI broth to achieve an initial density of 1 × 10^6^ CFU/mL. PIN was added to the bacterial suspension at final concentrations of 4, 16, and 64 μg/mL. The cultures treated with DMSO (0 μg/mL) served as the negative control. All samples were incubated anaerobically at 37 °C. A microplate reader (SpectraMax iD3; Molecular Devices, San Jose, CA, United States) was used to record the optical density at 600 nm (OD600) every hour throughout a 24-h period.

### 2.4 Biofilm formation assay

*S. mutans* suspensions were diluted to 1 × 10^6^ CFU/mL in BHIS medium and co-cultured with different concentrations of PIN (4, 16 μg/mL) in 96-well plates at 37 °C anaerobically for 24 h. DMSO-treated groups were set as negative controls. After incubation, the supernatant was removed, and the wells were gently rinsed three times with sterile PBS. The biofilms were fixed with 4% paraformaldehyde, stained with 0.1% crystal violet, and washed again to remove excess stain. The bound crystal violet was dissolved in absolute ethanol following air-drying. A microplate reader (SpectraMax iD3; Molecular Devices, San Jose, CA, United States) was used to measure the optical density at 575 nm (OD575). To exclude the confounding effect of bacterial growth inhibition at high compound concentrations, CFU enumeration was performed synchronously with the detection of biofilm formation. Treated biofilms were gently rinsed with sterile PBS to remove planktonic cells, resuspended, and fully homogenized by vigorous vortexing. The bacterial suspensions were serially diluted 10-fold, plated on BHI agar, and incubated anaerobically at 37 °C for 24 h for viable colony counting. All raw phenotypic values were normalized to the corresponding CFU counts of each sample to correct for variations in bacterial biomass.

### 2.5 Quantification of water-insoluble extracellular polysaccharides

The anthrone-sulfuric acid method was used to measure the amount of water-insoluble EPS produced in *S. mutans* biofilms following 24 h treatment with different concentrations of PIN. Planktonic bacteria and residual culture medium were thoroughly removed via gentle PBS rinsing. Biofilms were collected in 1.5 mL centrifuge tubes with 1 mL of 0.4 M NaOH. The supernatant was collected and combined with the anthrone-sulfuric acid reagent, heated for 6 min at 96 °C, then immediately cooled on ice. The absorbance value of each sample was detected at 625 nm (OD625) using the aforementioned microplate reader. Each treatment group was normalized to the corresponding CFU counts, as described in Section 2.4. The relative EPS content in each PIN-treated group was calculated by setting the DMSO control group as 100%.

### 2.6 Live/dead staining of biofilms

The LIVE/DEAD BacLight Bacterial Viability Kit (Thermo Fisher Scientific, Waltham, MA, United States) was used to determine the viability of *S. mutans* inside biofilms subjected to 24-h exposure to a range of PIN concentrations. The biofilm samples were carefully washed three times with sterile PBS in order to remove remaining medium and planktonic cells. Equal amounts of propidium iodide (PI) and SYTO 9 staining solutions were mixed in the dark and diluted 1,000-fold with sterile PBS. The prepared mixed staining solution was fully applied to the biofilm surfaces, and all samples were incubated for 15 min at room temperature under dark conditions. An inverted fluorescence microscope (Olympus CKX53, Japan) was used to view the samples after excess dye was gently rinsed away with PBS. The excitation/emission wavelength pairs were set as 480 nm/500 nm for SYTO 9 and 535 nm/617 nm for PI, respectively. The quantitative analysis was performed using Image J COMSTAT software. Values represent the means ± SD from three independent experiments (***P<0.001).

### 2.7 Scanning electron microscopy observation of biofilms

*S. mutans* biofilms were cultivated on sterile glass coverslips in 24-well plates and exposed to varying PIN concentrations for 24 h. The coverslips were gently washed with PBS and fixed with 2.5% glutaraldehyde (Solarbio, Beijing, China) overnight at 4 °C. Next, a gradient ethanol dehydration procedure was conducted for sample dehydration, followed by critical point drying with a K850 critical point dryer (Quorum Technologies, Lewes, United Kingdom). After drying, all specimens were sputter-coated with a thin layer of gold via a Smart Coater ion sputtering instrument (JEOL Ltd., Tokyo, Japan). A scanning electron microscope (SU8010; Hitachi High-Technologies, Tokyo, Japan) with an accelerating voltage of 5 kV and magnifications of 5,000 × and 20,000 × was used to examine the ultrastructure of the biofilms.

### 2.8 Bacterial adhesion assay

*S. mutans* mid-log-phase cells were collected, washed with sterile PBS, and then resuspended in fresh BHIS medium. DMSO-treated cultures were used as negative controls. PIN was added to the resulting bacterial solution at final concentrations of 4, 16 μg/mL. All samples were incubated for 24 h at 37 °C under anaerobic conditions. Following incubation, the supernatant containing planktonic bacteria was collected and its optical density at 600 nm (OD600) was measured to calculate the total planktonic cell density (ODt). The tubes were gently washed with sterile PBS to eliminate non-adherent bacteria. Bacteria adhering to the tube walls were re-suspended in sterile PBS through vigorous vortexing, and the optical density at 600 nm (OD600) was measured to obtain the adherent cell density (ODd). Each treatment group were normalized to the corresponding CFU counts, as described in Section 2.4.

### 2.9 Lactic acid measurement

PIN was applied to *S. mutans* biofilms at 4, 16 μg/mL. Cultures treated with DMSO served as the negative control. All samples were anaerobically incubated at 37 °C for 24 h. After incubation, the supernatant containing planktonic bacteria was discarded, and biofilm samples were rinsed twice with sterile PBS. The plates were then incubated under the same conditions for 2 h following the addition of buffered peptone water (BPW; Hopebio, China). Lactate concentrations were measured using the Lactic Acid Assay Kit (Jiancheng Bioengineering Institute, Nanjing, China) following the manufacturer’s instructions after the resultant supernatants were collected. The optical density at 530 nm (OD530) was measured using a microplate reader (SpectraMax iD3; Molecular Devices, San Jose, CA, United States), and each treatment group were normalized to the corresponding CFU counts, as described in Section 2.4.

### 2.10 Glycolytic pH drop assay

*S. mutans* was cultured in BHIS with PIN at 4, 16 μg/mL, while the negative control was DMSO. The initial pH of all bacterial suspensions was adjusted to 7.2 with 0.2 M KOH. The pH of the solution was then recorded every 30 min using a pH meter (FE28; Mettler Toledo, Greifensee, Switzerland).

### 2.11 qRT-PCR analysis

Biofilms of *S. mutans* were prepared as described in Section 2.4 and were treated with a sub-MIC concentration of PIN (16 μg/mL), and cultures treated with DMSO served as the control. Following anaerobic incubation at 37 °C for 24 h, the planktonic supernatants were removed, and the biofilms were rinsed twice with ice-cold sterile PBS. The biofilm cells were then harvested and lysed using 100 μL of 30 mg/mL lysozyme at 37 °C for 30 min. A bacterial RNA isolation kit (Foregene, Chengdu, China) was used to extract total RNA. Only samples with an A_260_/A_280_ ratio between 1.9 and 2.1 were utilized for subsequent experiments. After the purified RNA was dissolved in RNase-free water, 1 μg of total RNA was reverse-transcribed into first-strand cDNA using a reverse transcription kit (Foregene, Chengdu, China) with random hexamer primers. SYBR Green (Foregene, Chengdu, China) was used for qRT-PCR on a CFX96 Touch Real-Time PCR Detection System (Bio-Rad, Hercules, CA, United States). Tsingke Biotechnology (Beijing, China) synthesized primers for the target genes and the reference gene 16S rRNA, as shown in Table 1. The specificity of the amplified products was verified by melting curve analysis. The 2^−ΔΔCt^ method was utilized to determine relative gene expression utilizing the *S. mutans* 16S rRNA gene as the internal reference gene.

**Table 1:** Primers for qRT-PCR in this study.

Gene	Forward primer	Reverse primer	Product size (bp)
16S rRNA	AGCGTTGTCCGGATTTATTG	CTACGCATTTCACCGCTACA	156
*gtfB*	CACTATCGGCGGTTACGAAT	CAATTTGGAGCAAGTCAGCA	194
*gtfC*	GATGCTGCAAACTTCGAACA	TATTGACGCTGCGTTTCTTG	164
*gtfD*	TTGACGGTGTTCGTGTTGAT	AAAGCGATAGGCGCAGTTTA	219
*brpA*	CGTGAGGTCATCAGCAAGGTC	CGCTGTACCCCAAAAGTTTAGG	148
*dexA*	AGGGCTGACTGCTTCTGGAGT	AGTGCCAAGACTGACGCTTTG	142
*ftf*	ATTGGCGAACGGCGACTTACTC	CCTGCGACTTCATTACGATTGGTC	103
*spaP*	GACTTTGGTAATGGTTATGCATC	TTTGTATCAGCCGGATCAAGT	101
*srtA*	GAAGCTTCCTGTAATTGGCG	TTCATCGTTCCAGCACCATA	108
*vicK*	CACTTTACGCATTCGTTTTGCC	CGTTCTTCTTTTTCCTGTTCGGTC	102
*vicR*	CGCAGTGGCTGAGGAAAATG	ACCTGTGTGTGTCGCTAAGTGATG	157
*ldh*	AAAAACCAGGCGAAACTCGC	CTGAACGCGCATCAACATCA	255
*comD*	TTCCTGCAAACTCGATCATATAGG	TGCCAGTTCTGACTTGTTTAGGC	113
*comE*	TTCCTCTGATTGACCATTCTTCTG	GAGTTTATGCCCCTCACTTTTCAG	147

### 2.12 Rat dental caries model

All animal experimental protocols were approved by the Institutional Animal Care and Use Committee of North Sichuan Medical College (Approval No. NSMC2026007) and performed strictly following the ARRIVE 2.0 guidelines (Percie du Sert et al., 2020). Twenty SPF female Sprague–Dawley rats (21 days old, 70–80 g) were kept in individually ventilated cages with a 12-h light/dark cycle, 22 ± 2 °C, 50 ± 10% relative humidity, and unlimited access to Diet 2000 cariogenic diet (Trophic, Nantong, China) and 5% sucrose drinking water (Keyes, 1958). Body weight was recorded every 3 days. From days 1 to 3, each rat received drinking water containing 1 mg/mL ampicillin sodium (BioFroxx, Germany) daily to suppress the endogenous oral microbiota (Yu et al., 2024). From day 4 to day 10, rat oral cavities and tooth surfaces were inoculated with *S. mutans* suspension at a concentration of 1 × 10^8^ CFU/mL once a day. Specifically, 200 μL bacterial suspension was smeared on rat dentition via sterile cotton swabs, and another 150 μL was directly instilled into the oral cavity. Oral colonization was monitored weekly by swabbing the oral cavity and plating on selective Mitis Salivarius Bacitracin (MSB) agar plates throughout the experiment. To verify suppression of the endogenous oral microbiota, MSB plating was performed after antibiotic pretreatment and prior to *S. mutans* inoculation. Following inoculation, weekly MSB plating was continued to confirm sustained *S. mutans* colonization during the treatment period. After successful bacterial colonization, all rats were randomly allocated into four groups (*n* = 5) with different topical treatments, including 1% DMSO for the vehicle control group, sterile water for the untreated control group, 0.22% sodium fluoride (1,000 ppm F^−^) for the positive control group, and 512 μg/mL PIN for the experimental group. The PIN working solution for topical application was prepared in vehicle consisting of 1% DMSO. Test solutions were applied to the occlusal, buccal and lingual/palatal surfaces of the molars with sterile cotton swabs (1 min per application, twice daily), and excess solution was rinsed out with a blunt-tipped 5 mL syringe. Food and water were withheld for 2 h after each treatment. At the end of the 28-day treatment, all rats were euthanized by CO₂ inhalation. The maxillae and mandibles were collected for dental caries assessment using the Keyes scoring system, and the tongue, gingiva, spleen, liver, and kidneys were also harvested for subsequent histopathological analyses.

### 2.13 Keyes score assessment

Surrounding soft tissues were debrided to expose the dentition intact. The maxillae and mandibles were fixed in 4% paraformaldehyde for 24 h at 4 °C, then stained with 0.4% ammonium purpurate for 12 h at room temperature to visualize carious lesions. The maxillae and mandibles were hemisected sagittally along the mesiodistal axis of the molars, and the pits, fissures, and smooth surfaces were imaged under a stereomicroscope (SZ61-ZL; Olympus, Tokyo, Japan). Dental caries was graded using the Keyes scoring method (Keyes, 1958), which classified lesion severity into four categories: extensive dentinal (Dx), moderate dentinal (Dm), minor dentinal (Ds), and enamel only (E). Scoring was performed independently by two calibrated examiners who were blinded to the group allocation of all specimens. Inter-rater reliability was assessed by calculating the intraclass correlation coefficient (ICC).

### 2.14 Micro-CT analysis

The fixed mandibles were scanned on a micro-CT system (Pingsheng Medical Technology, Kunshan, China) at 70 kV, 200 μA, and a voxel size of 20 μm to characterize molar structure, mineral density, and lesion depth (Hoxie et al., 2023).

### 2.15 *In vivo* biosafety assessment

The extracted tongue, gingiva, spleen, liver, and kidney specimens were fixed in a 4% paraformaldehyde solution for 24 h (Zhao et al., 2026). The specimens were dehydrated using a graded ethanol series, paraffin-embedded, and sectioned. Sections were stained with hematoxylin and eosin (H&E), and histological morphology was examined under a light microscope (Eclipse Ci-L; Nikon, Tokyo, Japan).

### 2.16 Statistical analysis

The data are presented as mean ± standard deviation (SD). GraphPad Prism 10.1 (GraphPad Software, San Diego, CA, United States) was used to conduct statistical analyses. All quantitative data, were normalized to viable bacterial CFU counts to eliminate the interference of bacterial growth differences before statistical analysis. One-way ANOVA and Dunnett’s multiple comparisons test were used to examine group differences. qRT-PCR data were analyzed by unpaired *t*-tests with Holm-Šidák correction for multiple comparisons. Keyes caries scores at each lesion depth (E, Ds, Dm, and Dx) were analyzed separately using the Brown-Forsythe and Welch ANOVA, followed by Dunnett’s T3 post-hoc test for multiple comparisons. For all analyses, *p* < 0.05 was deemed statistically significant.

## 3 Results

### 3.1 Inhibition of planktonic growth of *Streptococcus mutans*

The chemical structure of PIN is shown in Figure 1A. As determined by the broth microdilution method, PIN exhibited an MIC of 32 μg/mL against planktonic *S. mutans* (Figure 1B). The MBC of PIN against *S. mutans* was determined to be 1,024 μg/mL (Figure 1C). According to the CLSI guidelines (Clinical and Laboratory Standards Institute, 2024), the calculated MBC/MIC ratio confirmed that PIN functions as a bacteriostatic rather than bactericidal agent under the tested conditions. The PIN concentrations (4 and 16 μg/mL) used in subsequent phenotypic assays were selected well below the bacteriostatic level. These non-lethal concentrations were suitable for investigating the anti-virulence regulatory effects of PIN, independent of its direct antibacterial killing activity.

**Figure 1:**
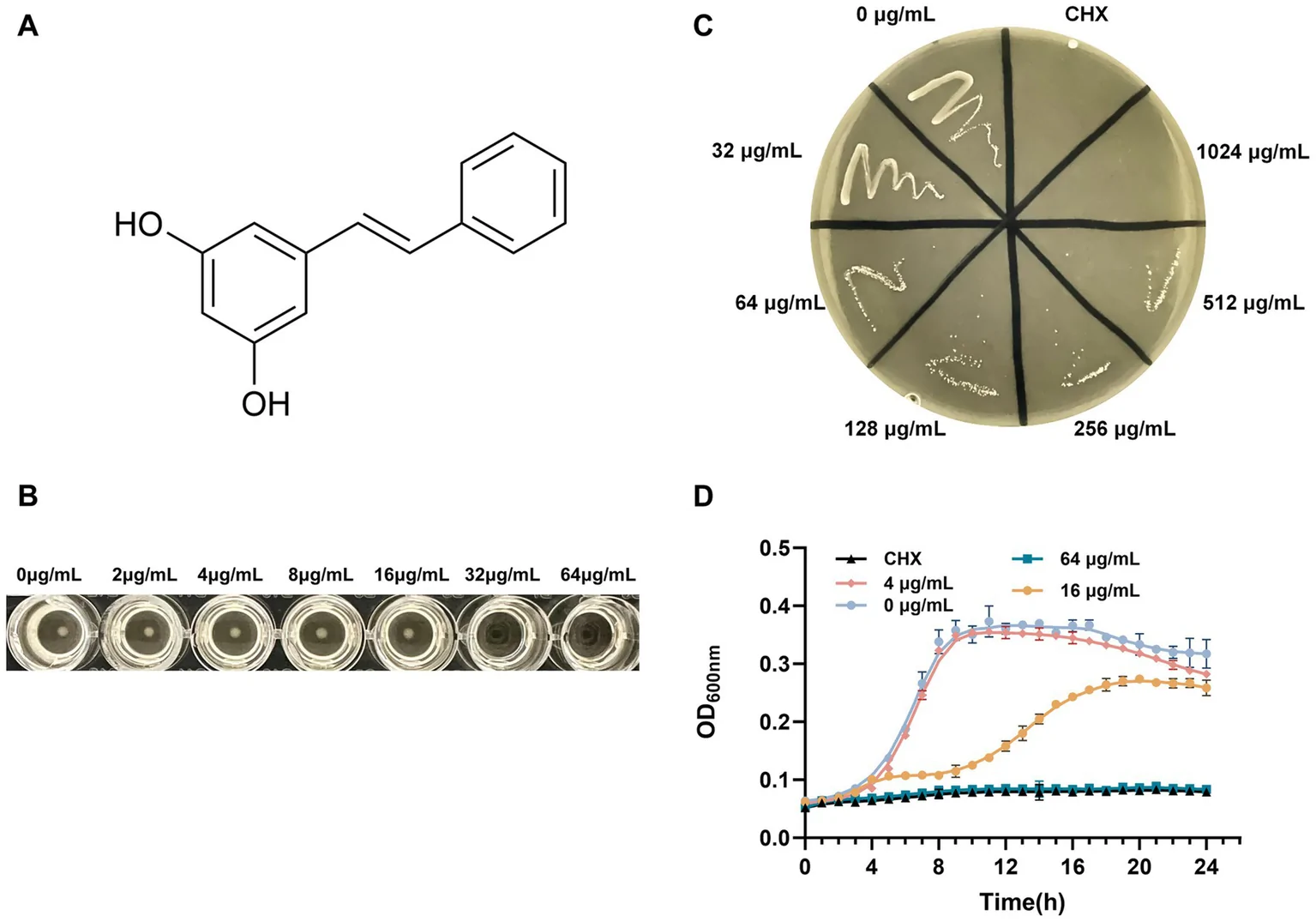
Effects of PIN on the planktonic growth of *S. mutans*. **(A)** Chemical structure of PIN. **(B)** Determination of the MIC of PIN against *S. mutans* using the broth microdilution method in a 96-well plate. The MIC was defined as the lowest concentration of PIN that completely inhibited visible bacterial growth. **(C)** MBC assay of PIN against *S. mutans*. **(D)** Growth curves of *S. mutans* monitored over 24 h by measuring the optical density at 600 nm (OD600) every hour. All values represent the means ± standard deviation (*n* = 3).

Time-dependent growth curves further confirmed the dose-dependent inhibitory effect of PIN on *S. mutans* proliferation. Specifically, upon exposure to sub-MIC concentrations (16 μg/mL), *S. mutans* exhibited a significantly extended lag phase along with a delayed logarithmic growth phase. Notably, 64 μg/mL PIN completely suppressed bacterial proliferation, an inhibitory effect comparable to that of the positive control CHX (Figure 1D).

### 3.2 Inhibition of biofilm formation and structure by PIN

*S. mutans* was treated with PIN at 4, 16 μg/mL to evaluate the effects on biofilm formation. Crystal violet staining demonstrated that PIN reduced total biofilm biomass in a dose-dependent manner, with the maximum inhibitory effect observed at 16 μg/mL, at which the inhibitory level of PIN on biofilm biomass was close to that of the positive control CHX (Figure 2A). The anthrone–sulfuric acid method was used to quantify water-insoluble EPS since it is essential to the formation of *S. mutans* biofilm. PIN reduced the production of water-insoluble EPS in a dose-dependent manner, with the greatest reduction observed at 16 μg/mL, and its inhibitory effect on EPS synthesis showed a similar trend to CHX (Figure 2B).

**Figure 2:**
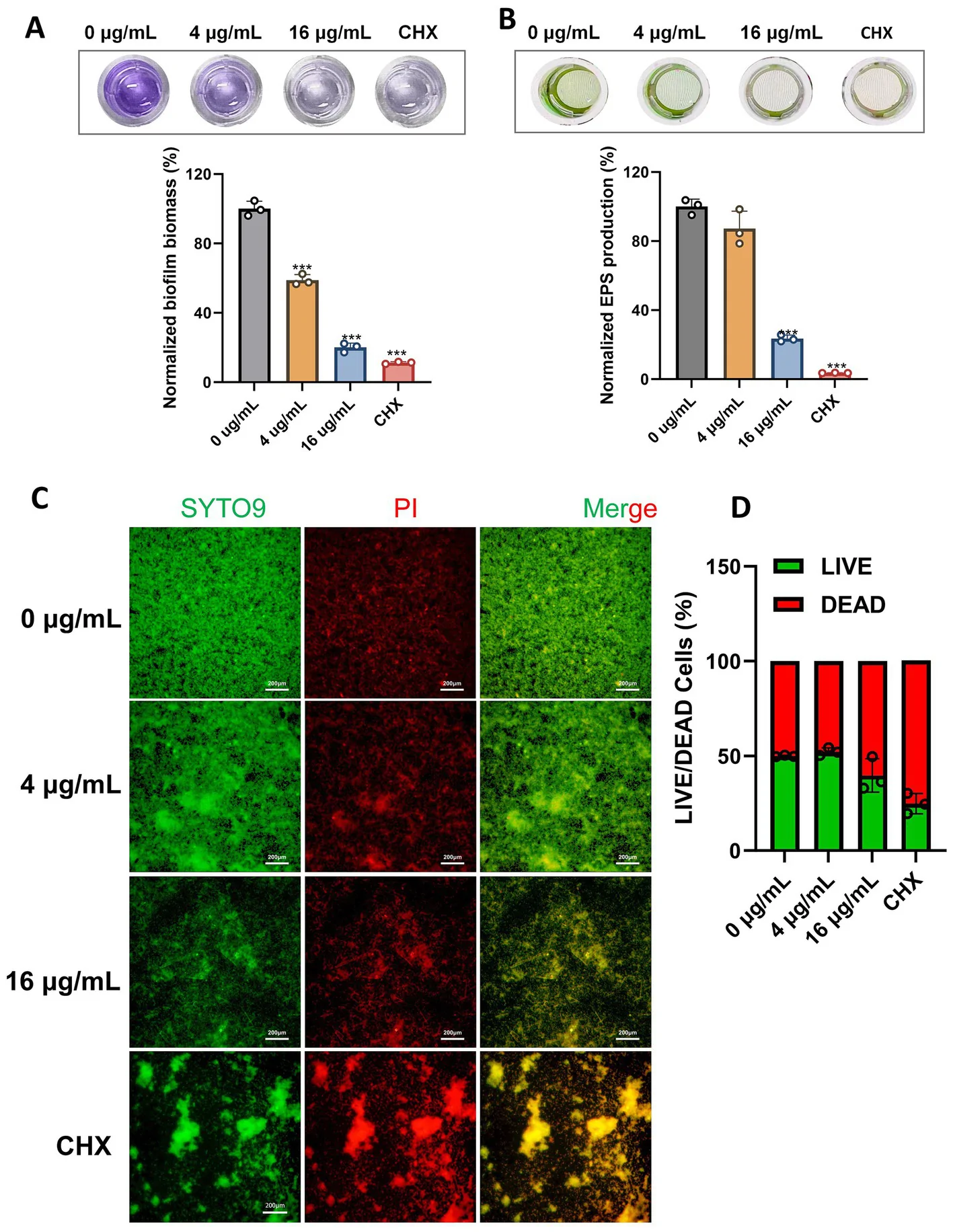
Effects of PIN on biofilm formation and architecture of *S. mutans*. **(A)** Crystal violet staining of *S. mutans* biofilms after treatment with PIN for 24 h. Quantification of biofilm biomass measured the optical density at 575 nm (OD575). **(B)** EPS production quantified by the anthrone-sulfuric acid method. **(C,D)** Bacterial LIVE/DEAD staining and corresponding semi-quantitative results. Representative fluorescence images of live/dead-stained *S. mutans* biofilms. Live cells were stained green with SYTO 9, and dead cells were stained red with PI (Scale bar: 500 μm). All values represent the means ± standard deviation (*n* = 3) (ns indicates no significant difference, ** *p* < 0.01, *** *p* < 0.001, compared with the control).

Biofilms formed after 24 h of incubation with different concentrations of PIN were observed using inverted fluorescence microscopy. SYTO 9/PI dual staining was used to assess biofilm integrity and distinguish between cells with intact membranes (live, stained green by SYTO 9) and those with damaged membranes (dead, stained red by PI). Fluorescence images showed that the negative control group’s green fluorescence intensity was markedly stronger than that of the other groups, showing a dense green structure. In contrast, the green fluorescence in the PIN-treated groups was markedly diminished, and the degree of biofilm disruption was similar to that of the positive control CHX. The live/dead staining assay demonstrated that, in the 16 μg/mL PIN group, the total biofilm mass was significantly reduced and its structural integrity was severely disrupted, yet unlike the CHX group with abundant red dead cells, a certain number of viable bacteria remained in the PIN group. Collectively, these results verified that PIN potently suppressed biofilm formation and biomass accumulation in *S. mutans* (Figure 2C). Figure 2D shows the quantitative results of LIVE/DEAD bacterial staining. Importantly, a proportion of bacteria remained viable (displaying green fluorescence) even at high PIN concentrations. These observations further indicate that PIN effectively disrupted the structural integrity and biomass of *S. mutans* biofilms.

To visually evaluate the effect of PIN on the three-dimensional ultrastructure of *S. mutans* biofilms, SEM was utilized for ultrastructural observation (Figure 3). In the control group, *S. mutans* formed a mature, dense, and multilayered biofilm network, wherein bacterial cells were tightly embedded within abundant EPS. However, following treatment with 4 and 16 μg/mL PIN, the three-dimensional structure of the biofilm became notably loose and disorganized. EPS production was substantially reduced, leaving the bacterial cells directly exposed and severely impairing the intercellular adhesion network, and the trend of biofilm disruption in the 16 μg/mL PIN group was similar to that of the positive control CHX. Overall, these images confirm that PIN disrupts the structural integrity of *S. mutans* biofilms.

**Figure 3:**
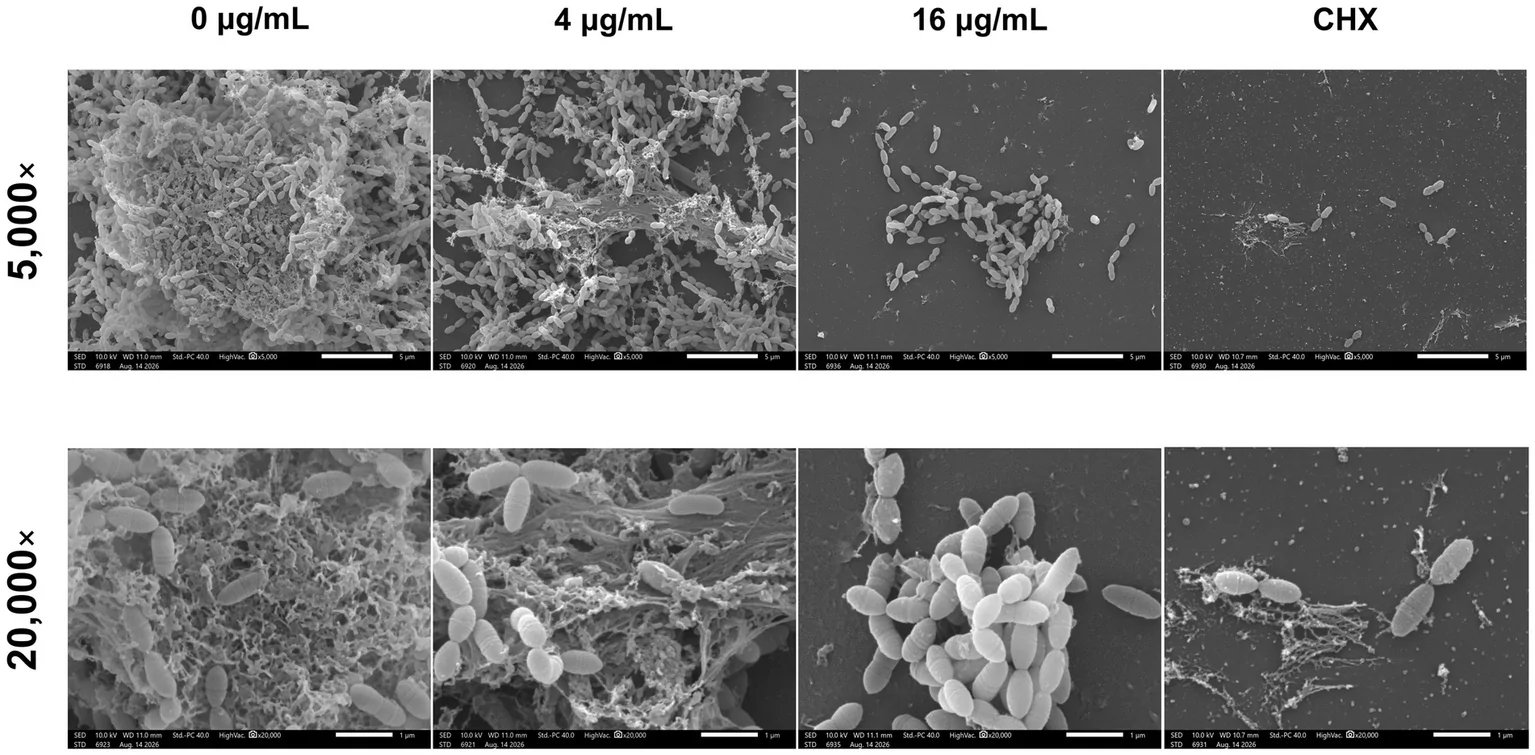
SEM images of *S. mutans* biofilms after treatment with PIN. *S. mutans* biofilms were treated with PIN for 24 h. The morphology of biofilms was observed by SEM at a magnification of 5,000 × and 20,000×. The control group displayed dense biofilm structures with abundant extracellular matrix, whereas PIN-treated groups exhibited sparse bacterial distribution and disrupted biofilm architecture in a concentration-dependent manner (Scale bar: 1 μm).

### 3.3 Reduction of adhesion and lactic acid production

Following confirmation that PIN disrupts *S. mutans* biofilms, further experiments were performed to examine its impacts on key cariogenic phenotypes. PIN decreased the adhesion rate of *S. mutans* in a dose-dependent manner, and 16 μg/mL PIN exhibited a similar inhibitory trend on bacterial adhesion to the positive control CHX (Figure 4A). Lactic-acid measurement assays demonstrated that 16 μg/mL PIN significantly suppressed lactic acid synthesis by the bacterium (Figure 4B).

**Figure 4:**
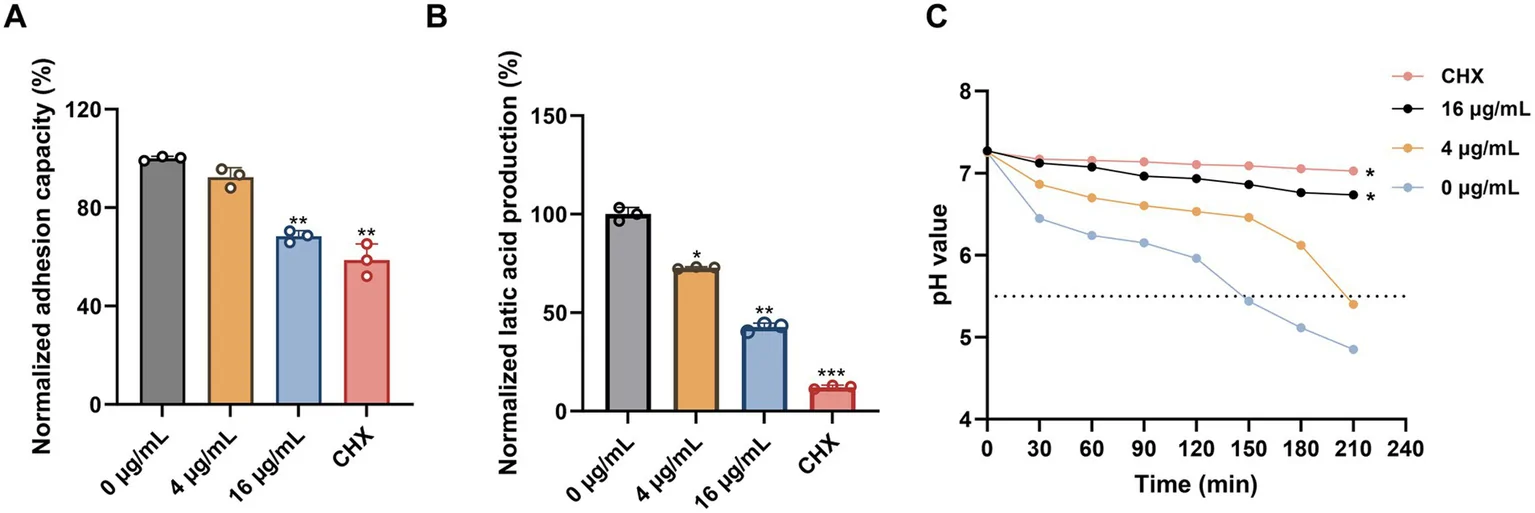
Effects of PIN on the cariogenic virulence factors of *S. mutans*. **(A)** Bacterial adhesion rate of *S. mutans* after treatment with PIN for 24 h. **(B)** Lactic acid production of *S. mutans* biofilms after PIN treatment. **(C)** Glycolytic pH drop curves of *S. mutans* suspensions monitored every 30 min for 4 h. All values represent the means ± standard deviation (*n* = 3) (* *p* < 0.05, ** *p* < 0.01, *** *p* < 0.001, compared with the control).

Enamel demineralization typically occurs at a pH below 5.5. While the microenvironment of the control group underwent rapid acidification, PIN dose-dependently suppressed acid generation via glycolysis. Consequently, the treatment effectively postponed the time at which pH dropped to the demineralization threshold, and 16 μg/mL PIN displayed comparable overall performance with CHX in retarding microenvironmental acidification (Figure 4C).

### 3.4 Downregulation of genes related to cariogenic virulence

Quantitative real-time PCR analysis was performed to characterize the transcriptional responses of major cariogenic virulence genes in *S. mutans* following sub-MIC PIN exposure. The *gtfB, gtfC, gtfD* and *ftf* genes are essential for extracellular polysaccharide synthesis and biofilm structural stabilization. In line with the reduced EPS synthesis and biofilm formation observed in phenotypic experiments, 16 μg/mL PIN significantly downregulated the transcription of these four genes (Figure 5A). Notably, at the transcriptional level, PIN treatment was associated with decreased transcription of the quorum-sensing two-component system (TCS) genes c*omD/comE* and the virulence-regulating TCS genes *vicK/vicR* (Figure 5B). In addition to TCS modulation, two adhesion-associated genes, *spaP* (encoding a surface adhesin for initial bacterial attachment) and *srtA* (encoding sortase A for surface protein anchoring), exhibited obvious transcriptional suppression after PIN intervention (Figure 5C). Consistently, reduced transcript levels of multiple functional genes associated with biofilm formation and bacterial cariogenicity, including the acid tolerance and biofilm-related gene *brpA*, acid production-related *ldh*, and dextran metabolism-related *dexA* (Figure 5D). Taken together, these transcriptional alterations indicate that PIN may affect the core components of the VicRK–ComDE regulatory system and downstream virulence-associated gene networks at the transcriptional level, although direct molecular targeting requires further functional validation.

**Figure 5:**
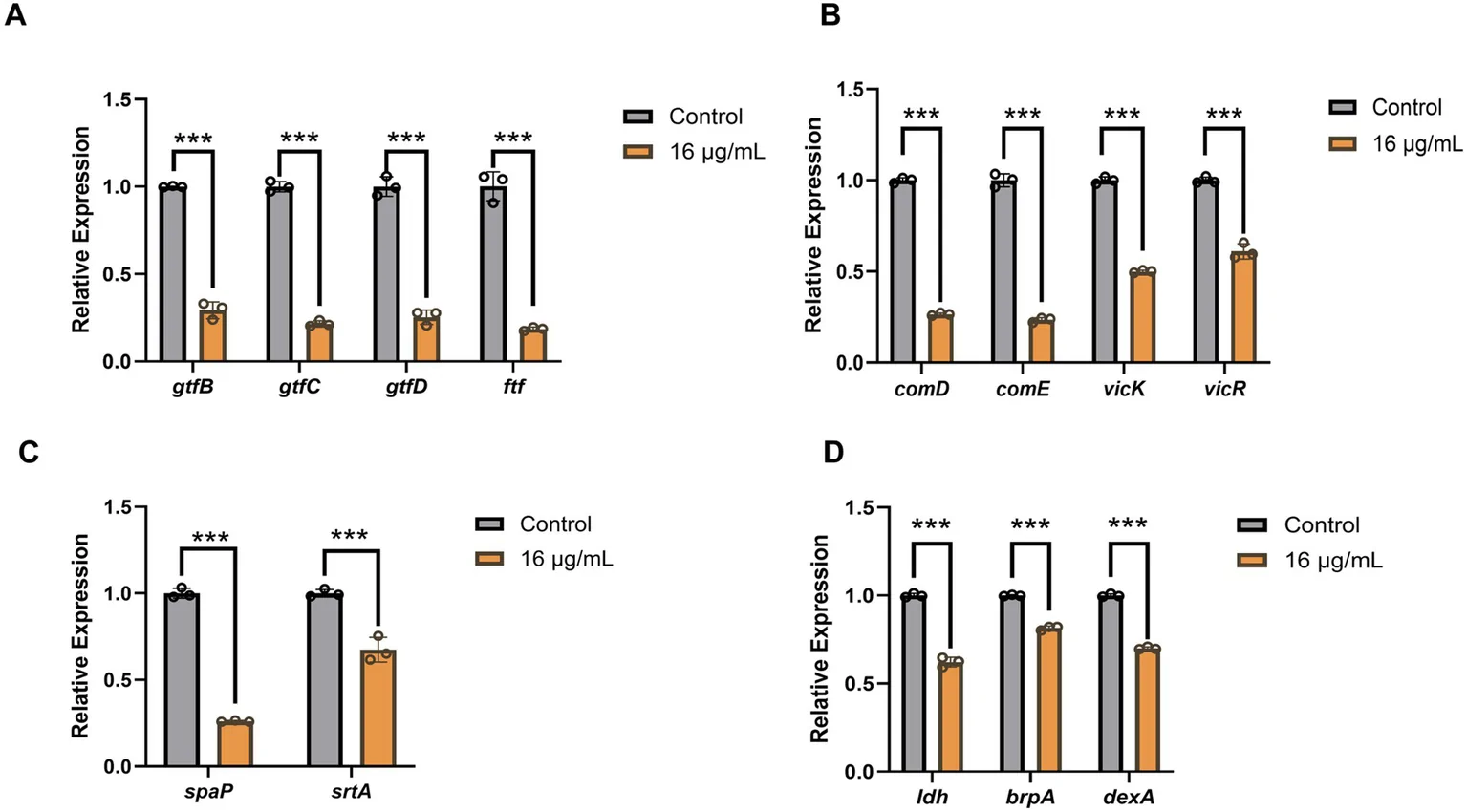
qRT-PCR analysis of cariogenic virulence-associated genes in *S. mutans* biofilms after treatment with PIN at 1/2 × MIC. **(A)** Exopolysaccharide synthesis genes: *gtfB*, *gtfC*, *gtfD*, and *ftf*. **(B)** Quorum sensing and two-component signal transduction genes: *comD*, *comE*, *vicK*, and *vicR*. **(C)** Adhesion and surface protein anchoring genes: *spaP* and *srtA*. **(D)** Acid production and biofilm regulation genes: *ldh*, *brpA*, and *dexA*. All values represent the means ± standard deviation (*n* = 3). Statistical significance was determined by unpaired *t*-tests with Holm-Šidák correction for multiple comparisons (* *p* < 0.05, ** *p* < 0.01, *** *p* < 0.001, compared with the control).

### 3.5 *In vivo* anti-cariogenic efficacy

A rat dental caries model was established to evaluate the *in vivo* anti-cariogenic efficacy of PIN (Figure 6A). All rats remained healthy throughout the 4-week experimental period, with no mortality or abnormal body-weight changes observed (Figure 6B). Quantitative assessment using the Keyes scoring system further quantified the extent of carious lesions. For superficial enamel lesions (E), no statistically significant difference in caries scores was observed between the PIN-treated group and the control groups under the current experimental conditions. However, as caries progressed into the dentin, the PIN-treated group exhibited significantly lower scores for slight (Ds), moderate (Dm), and extensive (Dx) dentinal lesions than the untreated control and DMSO vehicle groups (*p* < 0.05). The DMSO vehicle group exhibited typical severe enamel and dentin demineralization, and comparative analysis confirmed no significant differences in Keyes scores relative to the untreated control group. ns for all caries grades (Figure 6C). Molars stained with ammonium purpurate solution showed structural destruction and pronounced staining within the pits and fissures in the untreated control group. Consistently, the 1% DMSO vehicle control group also exhibited typical cariogenic lesions with obvious structural damage and deep staining, indicating that 1% DMSO itself had no anti-cariogenic activity and did not introduce additional interference to the experiment. By contrast, topical application of 512 μg/mL PIN markedly preserved tooth integrity, conferring protection comparable to that of the positive control (1,000 ppm NaF) (Figure 6D).

**Figure 6:**
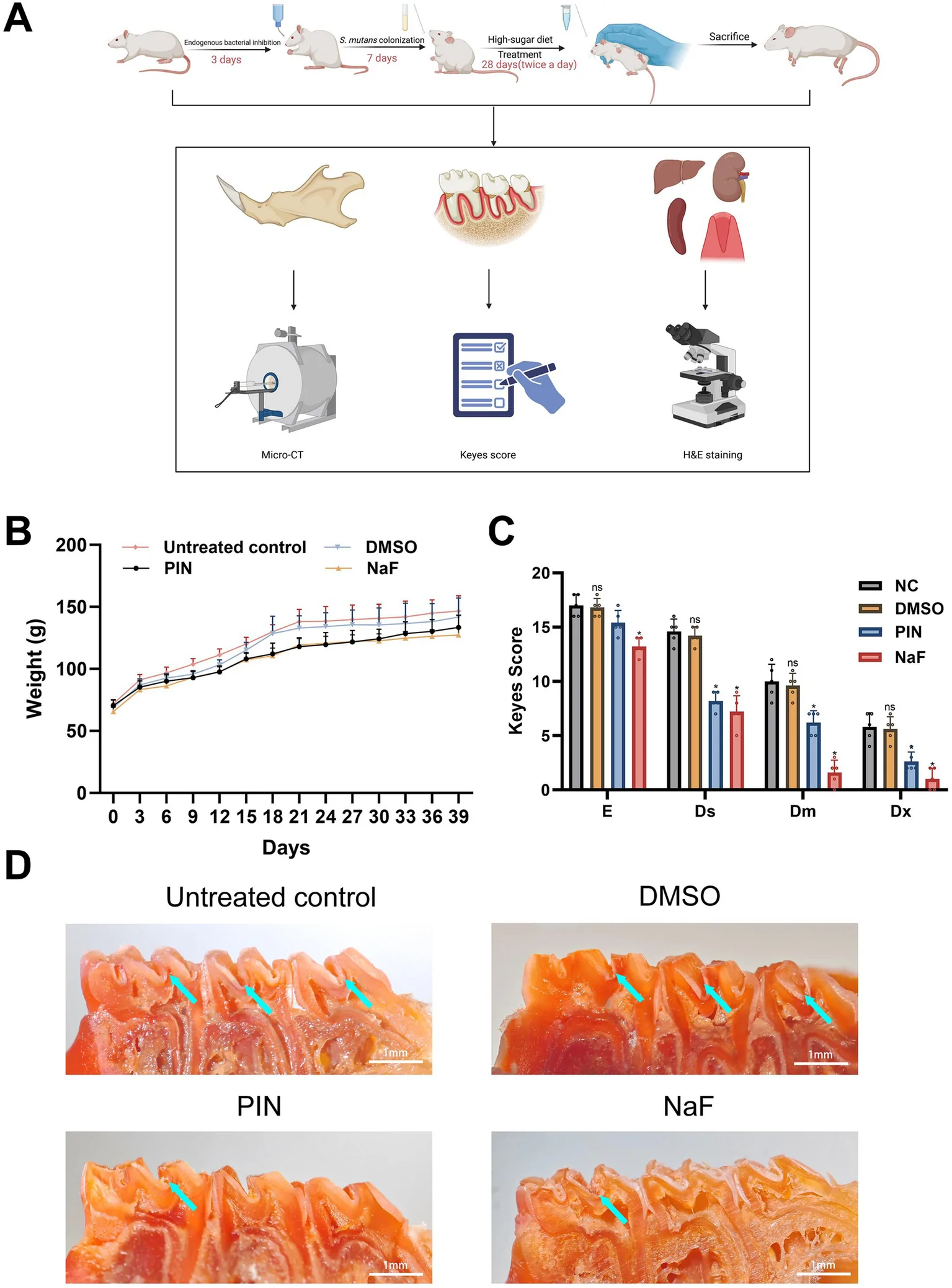
Effects of PIN on dental caries development in a rat model. **(A)** Schematic diagram of the *in vivo* experimental procedure. Rats were randomly divided into four groups (*n* = 5): untreated control (sterile water), positive control (1,000 ppm NaF), DMSO (1%) and PIN (512 μg/mL), and treated topically twice daily for 28 days. **(B)** Body weight changes of rats during the 4-week treatment period. **(C)** Quantification of caries severity using the Keyes scoring system: lesions confined to enamel **(E)**, slight dentinal lesions (Ds), moderate dentinal lesions (Dm), and extensive dentinal lesions (Dx). **(D)** Representative images of Keyes scoring. Blue arrows indicate carious lesions (Scale bar: 1 mm). All values represent the means ± standard deviation (*n* = 5) (ns indicates no significant difference, * *p* < 0.05, *** *p* < 0.001, **** *p* < 0.0001).

Micro-CT analysis demonstrated that the untreated control group exhibited extensive enamel demineralization and cavitated lesions within the deep fissures and interproximal surfaces, accompanied by underlying dentinal demineralization. The DMSO vehicle group also displayed severe enamel and dentin demineralization, with an overall lesion trend similar to the untreated control group. In the NaF group, typical subsurface demineralized zones were observed beneath a relatively intact, mineralized surface layer. Notably, the group treated with 512 μg/mL PIN only presented mild surface demineralization in deep grooves when compared with the control group, indicating that PIN could efficiently inhibit the progression of deep caries (Figure 7). Overall, these *in vivo* findings indicate that PIN shows anti-cariogenic effects under the tested experimental conditions.

**Figure 7:**
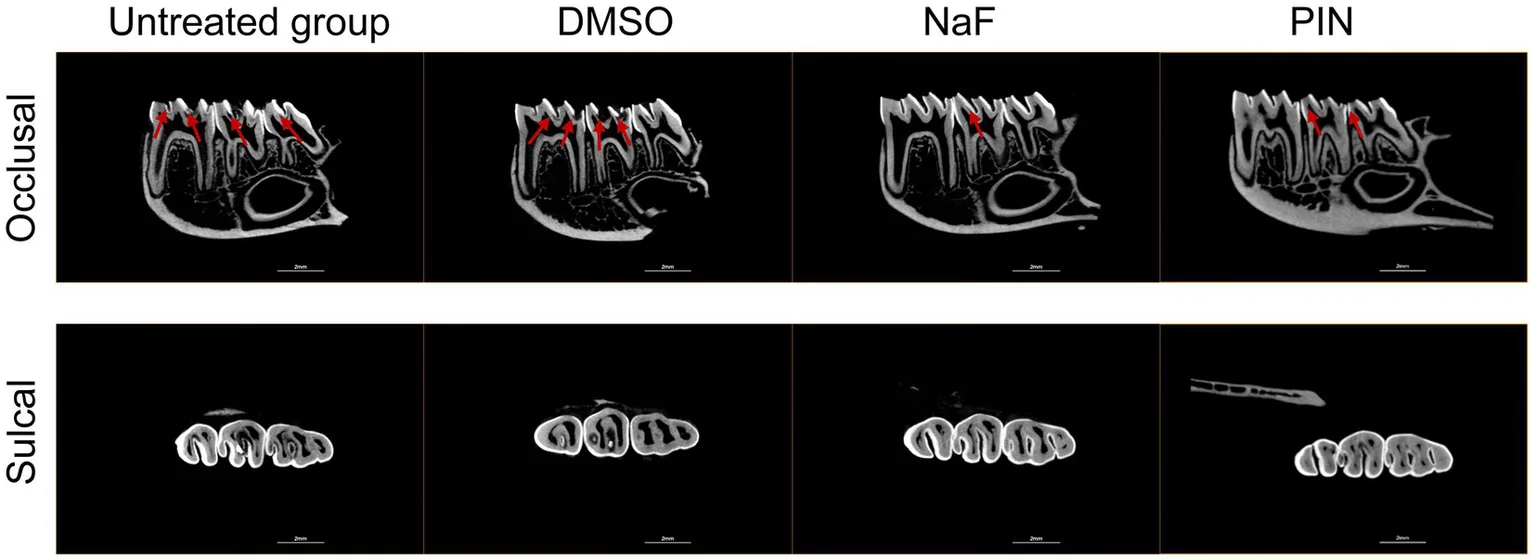
Representative micro-computed tomography (micro-CT) images of mandibular molars from rats with induced dental caries. Two sectional planes (occlusal and sulcal) were displayed across four experimental groups: untreated group, DMSO vehicle control, NaF positive control, and pinosylvin (PIN) treatment group. Red arrows indicate the location of carious lesions. Scale bar: 2 mm.

### 3.6 *In vivo* biosafety

Histopathological examination of major visceral organs and adjacent oral mucosa was conducted to assess the *in vivo* biosafety of PIN. Tissues from the PIN-treated group maintained normal cellular morphology and histological structure, according to H&E staining (Figure 8). No obvious pathological alterations were observed in comparison with the untreated control, DMSO group and NaF groups. Collectively, these data demonstrate that PIN shows favorable biocompatibility *in vivo*.

**Figure 8:**
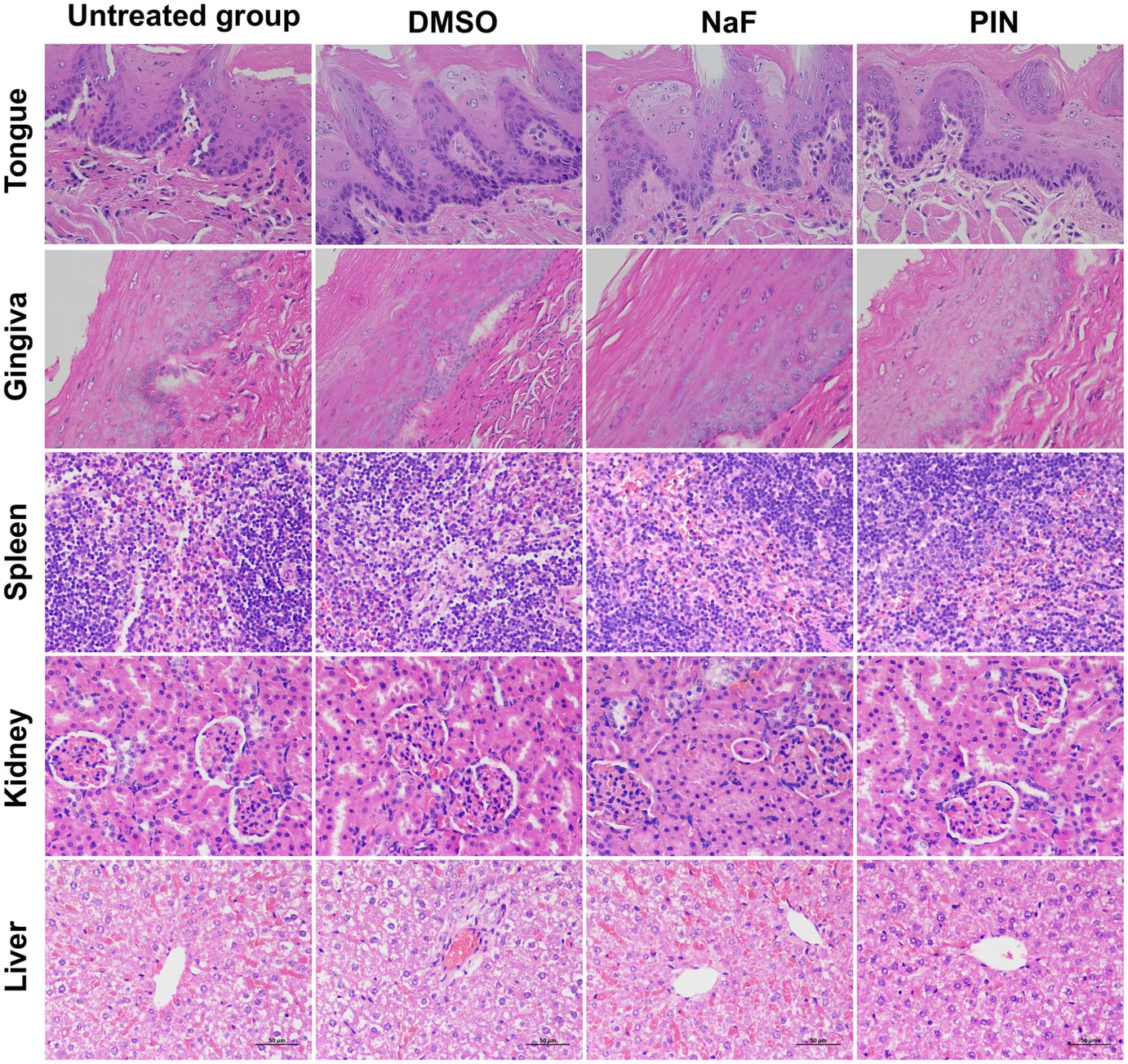
Representative photomicrographs of hematoxylin and eosin (H&E)-stained tissue sections from caries-challenged rats treated with vehicle (DMSO), NaF, or PIN, with untreated rats serving as the control. Five tissues were examined: oral tissues (tongue and gingiva) and major visceral organs (spleen, kidney and liver). Scale bar: 50 μm.

## 4 Discussion

Dental caries remains a major global public health burden. Acidogenesis, biofilm formation, and bacterial adhesion mediated by *S. mutans* have been identified as the primary drivers of cariogenic microenvironment development (Pitts et al., 2017). Given the limitations of current anti-caries strategies, natural bioactive compounds are widely investigated for their anti-cariogenic properties due to their diverse biological functions (Gloria-Garza et al., 2025). This work demonstrates that PIN, a natural stilbenoid polyphenolic compound, reduces acidogenicity, biofilm formation, and adhesion of *S. mutans* under *in vitro* experimental conditions. In addition, observations from a rat caries model show anti-cariogenic effects and favorable *in vivo* biocompatibility of PIN.

Conventional anti-caries agents have certain limitations, creating a demand for efficient and safe alternatives. Fluorides only facilitate dental remineralization, and prolonged use may induce fluoride resistance (Liao et al., 2017). Although chlorhexidine exhibits potent antibacterial activity, tooth discoloration, taste disturbance and the potential risk of bacterial resistance restrict its clinical application to short-term intervention (James et al., 2017). Natural compounds have attracted considerable attention as research subjects for oral anti-caries agents. Nevertheless, certain natural compounds trigger adverse effects that hinder practical application. For example, resveratrol exhibits dose-dependent inhibitory effects on *S. mutans*. Inhibitory activity can be observed at 50 μg/mL, and relatively high concentrations (200–400 μg/mL) are required to achieve robust suppression of its cariogenic phenotypes (Li J. et al., 2020). In addition, curcumin causes obvious tooth discoloration, whereas eugenol possesses antimicrobial activity against oral pathogens yet may elicit oral irritation and a burning sensation (Dinu et al., 2024). In contrast, PIN effectively inhibits the cariogenic virulence of *S. mutans* at 16 μg/mL. Meanwhile, under *in vivo* experimental conditions, PIN does not induce tooth discoloration or mucosal irritation, such adverse mucosal reactions. Even so, PIN also has inherent limitations, in that low water solubility in the oral environment restricts its sustained local bioavailability, and it is readily washed away by saliva, resulting in a short oral residence time. Future work will adopt hydrogel-based delivery systems to improve the oral compatibility of PIN and extend its oral residence time.

EPS synthesized by Gtfs constitute the structural scaffold of cariogenic biofilms, serving as a central determinant of the virulence of *S. mutans* (Zhang et al., 2021). Morphological characterizations via SEM and inverted fluorescence microscopy, combined with EPS quantification using the anthrone-sulfuric acid colorimetric assay, collectively demonstrated that PIN inhibited EPS biosynthesis in a concentration-dependent fashion. Consequently, biofilm architecture became loose, and bacterial cells lost physical protection. Beyond biofilm scaffolding, acidogenesis and adhesion are also recognized as critical virulence determinants leading to enamel demineralization (Shanmugam et al., 2020; Ma et al., 2022). Consistently, glycolysis-driven pH decline was significantly delayed, absolute lactate production was reduced, and the adhesion rate of *S. mutans* was substantially suppressed following PIN treatment. It should be noted that our adhesion assessment was performed using polystyrene microplates instead of saliva-coated enamel or hydroxyapatite substrates. This experimental setup was selected to align with the 24-h incubation design used for our biofilm and virulence assays and enabled simultaneous measurement of multiple phenotypes within the same batch of cultures. Nevertheless, polystyrene cannot mimic the physiological substrate that *S. mutans* encounters during caries formation (Azeredo et al., 2017). The inhibitory magnitude measured on polystyrene surfaces may not be equivalent to the effect on saliva-coated tooth surfaces, which limits the generalisabilityof these adhesion results to authentic *in vivo* tooth-surface adhesion. Future experiments using saliva-coated enamel or hydroxyapatite substrates are therefore needed to validate PIN’s anti-adhesive activity under more physiologically relevant conditions.

At the molecular level, a sophisticated interconnected genetic network modulates the pathogenic potential of *S. mutans* (Zheng et al., 2023). EPS is predominantly synthesized by Gtfs and fructosyltransferases (Ftfs) (Atta et al., 2024). In this system, GtfD produces water-soluble glucans, GtfC produces both soluble and insoluble glucans, and GtfB produces insoluble glucans (Lemos et al., 2019). In the present study, PIN treatment was associated with significant reductions in the transcript levels of *gtfB, gtfC*, and *gtfD*, an observation made alongside reduced EPS biosynthesis in phenotypic assays. In the present study, sub-minimum inhibitory concentration (sub-MIC) PIN did not cause apparent lethal damage to *S. mutans* but altered transcript levels of genes associated with bacterial TCS. These systems are known to coordinate many virulence traits and enable bacteria to sense and dynamically respond to environmental fluctuations. Acting as central regulatory hubs, they govern EPS synthesis, bacterial adhesion, and stress adaptation. Typically composed of a membrane-anchored histidine kinase and a cytoplasmic response regulator, the pair orchestrates transcriptional reprogramming of virulence-associated pathways. Through this cascade, bacteria adjust their physiological properties to survive and establish colonization within the oral cavity (Senadheera et al., 2005; Senadheera et al., 2012; Qiu et al., 2025). Notably, the current qPCR results cannot distinguish whether PIN regulates TCS pathways through direct protein interaction or indirect modulation of bacterial physiological homeostasis. Further functional verification (such as protein-ligand binding assay, kinase activity detection, gene complementation experiment) is required to clarify the precise molecular mechanism in follow-up research.

For early oral colonization, transcriptional levels of the surface adhesin gene *spaP* and its anchoring enzyme coding gene *srtA* were drastically decreased (Hu et al., 2021; Zhou Y. et al., 2025). Furthermore, parallel downregulation of lactate dehydrogenase (*ldh*) and the pivotal stress-response gene *brpA* was observed. These genes have been linked to bacterial acid production and low-pH defense responses (Boisen et al., 2023). Notably, the dextranase-encoding gene *dexA* was also significantly transcriptionally suppressed. Although DexA degrades water-soluble glucans, its proper function is required for matrix remodeling; transcriptional shifts in *dexA* may have consequences for biofilm aggregation and structural assembly, as previously described (Yang et al., 2019).

*In vivo* studies are crucial to evaluate the anti-cariogenic efficacy and biosafety of PIN. In the *in vivo* experiment of this study, 512 μg/mL PIN was selected for topical intervention. This concentration corresponds to half of the MBC and falls within the commonly adopted dosage range for topical oral anti-virulence and anti-biofilm studies using animal models (Yu et al., 2024; Chi et al., 2025). Considering salivary clearance and biofilm barriers within the oral microenvironment, topically applied free compound are prone to loss and struggle to sustain sufficient local drug exposure (Horev et al., 2015). Therefore, a relatively high local dosage was adopted in this study. This dosage inhibits bacterial growth without complete bacterial eradication, which is suitable for exploring bacterial phenotypic modulation rather than bactericidal effects and matches the core aim of our work. Meanwhile, PIN at 512 μg/mL shows favorable biocompatibility with oral mucosa and causes no obvious irritation. Notably, only one concentration was tested *in vivo*, so a full dose–response relationship between PIN exposure and caries lesion severity could not be established. This represents one limitation of the present study. Follow-up animal work may include multiple dose groups to systematically characterize the dose-dependent anti-cariogenic effects of PIN and identify suitable experimental concentrations.

Micro-CT analysis revealed markedly less enamel demineralization in the PIN-treated group, with lesions confined to superficial pits and fissures, whereas extensive demineralization extending into dentin was documented in the untreated control group. Quantitative Keyes caries scoring further validated such dentin-protective effects. While PIN failed to fully block early superficial enamel lesions (grade E), it remarkably lowered both the prevalence and severity of dentinal caries lesions graded Ds, Dm and Dx (*p* < 0.05). Although NaF primarily promotes surface remineralization via the formation of fluorapatite, which explains its superior performance at the early enamel caries stage, its overall efficacy is somewhat constrained by a limited ability to inhibit mature biofilms (Cai et al., 2021; Han, 2021). By contrast, PIN directly dismantled the biofilm matrix and neutralized the acidogenic microenvironment. This mechanistic divergence suggests that potential combinatorial effects may be achieved through combinatorial application: PIN disrupts biofilm architecture, while low-dose NaF drives remineralization. This synergistic hypothesis warrants rigorous validation in future studies.

Based on the distinct experimental conditions, biological properties of DMSO, and solubility characteristics of PIN, different DMSO concentrations were applied for *in vitro* and *in vivo* experiments in this study (Tunçer Çağlayan and Gurbanov, 2024). For *in vitro* bacterial assays, continuous solvent exposure may interfere with bacterial gene expression, membrane characteristics and phenotypic profiles, thereby compromising experimental accuracy. To minimize solvent-derived effects, 0.5% DMSO was adopted for *in vitro* tests, which guaranteed sufficient PIN solubility while reducing unnecessary interference (Jo and Kim, 2024; Jo et al., 2026). In contrast, oral topical DMSO *in vivo* was rapidly diluted by saliva. This transient exposure causes few non-specific experimental alterations and induces no mucosal irritation. Therefore, 1% DMSO was used in the *in vivo* model to fully dissolve PIN and satisfy the requirements of local oral administration, and this concentration was selected with reference to previously reported similar caries studies (Ren et al., 2016; Zhou et al., 2023; Wu et al., 2024).

Biocompatibility is an important consideration when characterizing bioactive compounds. *In vivo* histopathological examination of rat oral mucosal tissues and vital visceral organs verified the favorable biological safety of PIN, with no detectable histopathological lesions or organ toxicity observed across all tested specimens. Furthermore, unlike certain antimicrobial peptides that may elicit immunogenic responses (Oliveira Júnior et al., 2025), PIN is a plant-derived polyphenol with well-documented anti-inflammatory, antioxidant, and anti-tumor biological activities (Periferakis et al., 2025). This favorable safety profile warrants further investigation of PIN using oral biological models.

Although these results are encouraging, it should be recognized that there are some limitations. First, *S. mutans* was the only cariogenic pathogen that was examined in this investigation. However, the human oral cavity hosts a sophisticated polymicrobial community featuring complex metabolic cooperation and competitive interspecies interactions (Hajishengallis et al., 2023). Given that dental caries is inherently a polymicrobial disease, single-species models cannot accurately replicate its pathogenesis (Lamont et al., 2018). Multi-species biofilm models incorporating *Streptococcus sanguinis*, *Streptococcus gordonii*, and *Streptococcus salivarius* (Ye et al., 2025) should be employed in future work to evaluate the ecological impact of PIN and to determine whether cariogenic taxa are selectively targeted while commensals are preserved. A formal *a priori* power analysis was not performed before animal randomization for this *in vivo* rat caries experiment. Therefore, the finding of no statistically significant difference for enamel (E) lesions observed under the present experimental conditions must be interpreted cautiously; this observation does not rule out the possibility that a treatment effect may become detectable with a larger sample size. Future confirmatory animal studies should perform predefined power calculations prior to animal randomization, to better assess the effects of compound on enamel caries. Furthermore, short-term adhesion assays on saliva-coated hydroxyapatite or enamel, which faithfully recapitulate initial bacterial-tooth surface contact, were not performed in the current study, which centered on comprehensive evaluation of persistent cariogenic traits of *S. mutans* under 24-h culture conditions. Subsequent research may incorporate these adhesion assays to further assess how PIN modulates the early adhesion process of cariogenic bacteria. As a polyphenolic compound, PIN exhibits low water solubility (Bakrim et al., 2022). Owing to the special oral environment. PIN is readily washed away by saliva, leading to unstable local compound retention, which limits its sustained local bioavailability. Without optimized delivery carriers, these drawbacks greatly restrict its utility in oral environments. Plant-derived exosome-like nanovesicles offer promising biocompatible carriers to retain active agents during mucosal delivery (Mu et al., 2023). This strategy is supported by studies on curcumin, where targeted delivery systems like pH-responsive nanoparticles, polymeric micelles, and liposomes have significantly mitigated comparable solubility and bioavailability challenges (Li L. et al., 2020). Therefore, follow-up experimental studies may explore mucoadhesive PIN nanoformulations to prolong intraoral retention and enhance biofilm penetration.

In conclusion, this study indicates that PIN could partially attenuate the cariogenicity of *S. mutans.* This activity is accompanied by suppression of acidogenesis, extracellular polysaccharide synthesis, and biofilm formation, together with altered transcription of key virulence-associated genes. With the preliminary anti-cariogenic effects and acceptable biocompatibility observed in the present study, PIN represents a natural bioactive molecule worthy of further mechanistic investigation.

## Data Availability

The raw data supporting the conclusions of this article will be made available by the authors.
